# The Diagnostic Accuracy of Neutrophil-to-Lymphocyte Ratio (NLR) Compared to C-Reactive Protein (CRP) in Patients with Acute Cholecystitis: A Systematic Review and Meta-Analysis

**DOI:** 10.3390/diagnostics16091363

**Published:** 2026-04-30

**Authors:** Raluca-Ioana Șerban, Isaic Alexandru, Cristi Tarta, Faur Flaviu-Ionut, Duta Ciprian, Alexandru Catalin Motofelea, Dobrescu Amadeus Emanuel

**Affiliations:** 1 Doctoral School, “Victor Babes” University of Medicine and Pharmacy, 300041 Timisoara, Romania; raluca.serban@umft.ro; 2 Department X of General Surgery, “Victor Babes” University of Medicine and Pharmacy, 300041 Timisoara, Romania; tarta.cristi@umft.ro (C.T.); flaviu.faur@umft.ro (F.F.-I.); duta.ciprian@umft.ro (D.C.); dobrescu.amadeus@umft.ro (D.A.E.); 3 Centre for Molecular Research in Nephrology and Vascular Disease (MOL-NEPHRO-VASC), “Victor Babes” University of Medicine and Pharmacy, 300041 Timisoara, Romania; alexandru.motofelea@umft.ro

**Keywords:** acute cholecystitis, neutrophil-to-lymphocyte ratio, C-reactive protein, diagnosis

## Abstract

**Background/Objectives:** Acute cholecystitis is associated with an increased risk of morbidity and mortality. Thus, early diagnosis and detection of complications are essential. Neutrophil-to-lymphocyte ratio (NLR) and C-reactive protein (CRP) are inflammatory biomarkers that could predict the diagnosis and complications of acute cholecystitis. Yet, the results of which biomarker has higher accuracy are inconsistent. **Objective**: To compare the accuracy of NLR and CRP in the diagnosis and prediction of complications in patients with acute cholecystitis. **Methods:** We searched PubMed, Scopus, and Web of Science in January 2026 for studies that compared both biomarkers (NLR and CRP) for the diagnosis and detection of complications or severity in patients with acute cholecystitis. We assessed the quality of the included studies using the QUADAS 2 tool. A bivariate meta-analysis was performed using R to compare the pooled diagnostic odds ratio (DOR) and the difference in sensitivity and specificity between both biomarkers. We used RevMan software to generate forest plots and summary receiver operating characteristic (SROC) curves using parameters calculated through R. **Results:** We included 15 studies in the systematic review, and 12 of them were included in the meta-analysis. The pooled data showed that NLR had higher accuracy in the diagnosis of acute cholecystitis compared to CRP, DOR 2.257 (95% CI 1.1, 4.633); however, there was no significant difference in sensitivity or specificity. There was no significant difference between NLR and CRP in detecting complications, including perforation, gangrene, and suppurative cholecystitis, DOR 1.100 (95% CI 0.817, 1.481). Meanwhile, NLR had similar sensitivity but lower specificity −0.050 (95% CI −0.090, −0.010) compared to CRP. NLR had lower overall accuracy in the detection of disease severity grades according to the Tokyo guidelines compared to CRP, DOR 0.170 (95% CI 0.081, 0.359), but higher specificity 0.230 (0.182, 0.279) compared to CRP. **Conclusions:** This systematic review and meta-analysis showed that NLR had higher diagnostic accuracy than CRP in patients with acute cholecystitis. However, both biomarkers had comparable sensitivity and specificity. Additionally, both biomarkers had comparable accuracy in detecting complications such as perforation, gangrene, or suppurative cholecystitis. CRP had higher overall accuracy in detecting disease severity according to the Tokyo guidelines. Thus, while NLR may be useful in diagnosing acute cholecystitis, CRP could be more effective in assessing disease severity. However, owing to the limited number of included studies, these findings should be interpreted with caution, and further studies are needed to confirm these results.

## 1. Introduction

Gallbladder and biliary tract diseases represent a major burden worldwide. Their prevalence has significantly increased by around 60% over the past years; this was associated with a significant increase in mortality rates [1]. These include cholangitis and acute cholecystitis. Acute cholecystitis is a condition characterized by gallbladder inflammation, occurring due to cystic duct obstruction by stones in 90% of patients. Nonetheless, it could occur without stones in patients with severe conditions [2,3,4]. Acute cholecystitis affects around 10% of patients with acute abdomen presenting to the emergency department [3]. It is associated with an increased risk of morbidity and mortality since it could be further deteriorated by local complications such as gangrene and perforation and systemic complications such as multiple organ failure. Thus, early diagnosis is important to reduce the incidence of complications and to choose the optimal treatment, while early assessment of the severity of the condition could reduce mortality rates [3,5].

The diagnosis of acute cholecystitis depends on imaging and local and systemic signs of inflammation [6]. These include elevated levels of C-reactive protein (CRP) or white blood cells (WBCs) as biomarkers of systemic inflammation. The Tokyo guidelines categorized patients with acute cholecystitis according to the severity of the condition into mild, moderate, and severe cases [6]. However, the detection of complicated cholecystitis through imaging or clinical examination and presenting symptoms could be challenging since clinical and imaging findings can be non-specific [7,8,9,10,11]. While having a high WBC count is considered a diagnostic criterion in moderate cases, as stated by the Tokyo guidelines, the utility of other markers of inflammation as diagnostic and prognostic markers is not yet established.

Acute cholecystitis is an inflammatory condition. Thus, it is associated with leukocytosis and neutrophilia [12]. The neutrophil-to-lymphocyte ratio is an inflammatory biomarker used to predict several conditions, such as sepsis [13]. Recently, Kler et al. found that NLR could be used to predict acute cholecystitis. Moreover, NLR was significantly higher in patients with severe cholecystitis [14]. Furthermore, CRP has been suggested to be useful in the prediction of complications and length of hospital stay [15].

Several studies assessed the utility of CRP and NLR for the diagnosis of acute cholecystitis as well as the detection of severity grades and complications. Yet, they had inconsistent findings [16,17,18,19,20,21]. For instance, while some studies showed that both biomarkers were comparable in detecting complicated cholecystitis [16,19], others showed that CRP had higher accuracy [17]. Thus, we conducted this systematic review and meta-analysis to compare the accuracy of NLR and CRP in the diagnosis and prediction of complications in patients with acute cholecystitis.

## 2. Materials and Methods

We performed this systematic review and meta-analysis following the Preferred Reporting Items for Systematic Reviews and Meta-Analyses (PRISMA) [22]. The PRISMA checklist is in the Supplementary Materials (Supplementary File S1). The study was prospectively registered in PROSPERO under registration number CRD420261321493.

### 2.1. Literature Search

We searched Web of Science, Scopus, and PubMed in January 2026 using the following keywords (“Acute cholecystitis” OR “acute calculous cholecystitis” OR “acute non-calculous cholecystitis OR “Complicated Acute Cholecystitis”) AND (“C-reactive protein” OR CRP) AND (“neutrophil-lymphocyte Ratio” OR NLR). The full search strategy is in the Supplementary File.

### 2.2. Eligibility Criteria and Study Selection

We included observational studies that included patients with acute cholecystitis who underwent blood tests for measuring both NLR and CRP, where the diagnostic accuracy of both modalities was compared in the diagnosis or prognosis of acute cholecystitis.

We excluded duplicates, reviews, studies not available in English, or where the full text could not be retrieved. Additionally, we excluded studies that did not provide separate data for patients with acute cholecystitis and studies that did not provide data for both modalities.

The results of the literature search were collected in an Excel sheet and screened through two stages. First, we screened the studies according to their title and abstract, and then we screened the full text of the retrieved studies.

### 2.3. Data Extraction and Quality Assessment

The authors extracted the data from the included studies using Excel sheets. The extracted data from the included studies were the general characteristics, baseline characteristics, and outcomes. The general characteristics of trials included the country, time of conduction, study design, and the sample size. The baseline characteristics of patients included age and gender. The outcomes involved the accuracy of the diagnosis or determination of complicated acute cholecystitis collected as true positive, true negative, false positive, and false negative values, or AUC values and their corresponding 95% CI.

The quality of the included studies was evaluated using the QUADAS-2 tool [23]. The tool involves four main domains and three applicability domains. These are patient selection in addition to their applicability, index test and its applicability, reference standard and its applicability, and flow and timing. Signaling questions for each domain were answered with Yes, No, or unclear. Accordingly, the domains were judged as low risk, unclear, or high risk of bias.

### 2.4. Statistical Analysis

We performed the analysis using RevMan 5.4 software and R software version 4.4.3. We performed a bivariate meta-analysis using a generalized linear mixed model to compare the diagnostic accuracy of NLR and CRP. This accounts for between-study heterogeneity and the within-study correlation between sensitivity and specificity. The heterogeneity was quantified using τ^2^ derived from the random effects structure model for sensitivity and specificity, along with the covariance and correlation between them. The likelihood ratio tests were used to assess whether the diagnostic accuracy differed significantly between the two biomarkers. Summary estimates of sensitivity and specificity were back-transformed to probability scales, and absolute differences along with the diagnostic odds ratios (DOR) were calculated with 95% confidence intervals. The summary receiver operating characteristic (SROC) plots were generated with RevMan, using the parameters calculated from R. Additionally, we conducted a sensitivity analysis to confirm the reliability of our findings. Studies with isolated zero cells (true positive, true negative, false positive, or false negative) were retained since model convergence was achieved.

## 3. Results

### 3.1. Search Results and Study Selection

**Figure 1:**
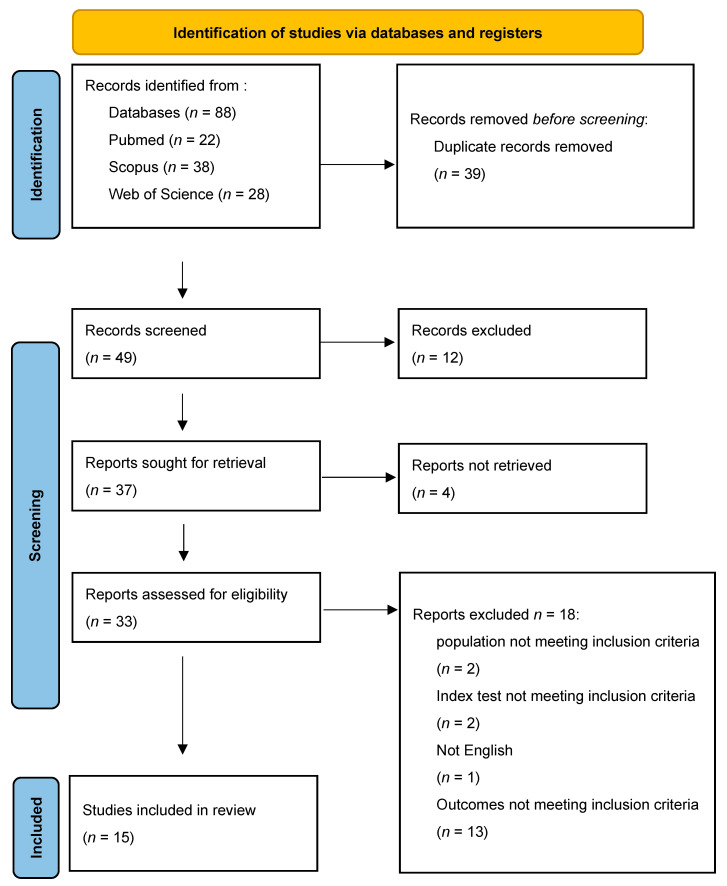
PRISMA flowchart of included studies.

The literature search retrieved 88 articles, of which 39 were duplicates (Figure 1). Title and abstract screening was performed on 49 articles, while 33 articles were screened according to their full text. Finally, 15 studies [16,17,18,19,20,21,24,25,26,27,28,29,30,31,32] were included in this systematic review, of which 12 [17,18,19,21,24,25,27,28,29,30,31,32] were meta-analyzed.

### 3.2. Characteristics of Included Studies

The summary and baseline characteristics of the included studies are shown in Table 1. Four studies were prospective, while eleven were retrospective. The sample size of the included studies ranged from 56 to 1352. Among the included studies, the data for the analysis were not fully reported in three studies [16,20,26]; thus, they were excluded from the analysis. Two studies assessed the role of NLR and CRP in the diagnosis of acute cholecystitis [25,29], ten studies investigated their role in predicting complications [17,19,20,21,24,26,27,28,30,32], while three studies investigated both [16,18,31]. The reference standard was histopathological examination of the gallbladder in 10 studies [16,17,19,21,24,26,27,29,31,32], while three studies assessed the clinical deterioration of patients according to the Tokyo guidelines [18,20,28]. One study used histopathology or Tokyo severity grades [30], while another study used clinical diagnosis according to the Tokyo guidelines as the reference standard [25]. The mean age of the participants ranged from 49 to 76, and the percentage of males ranged from 30.5% to 62.5%.

### 3.3. Quality Assessment

**Figure 2:**
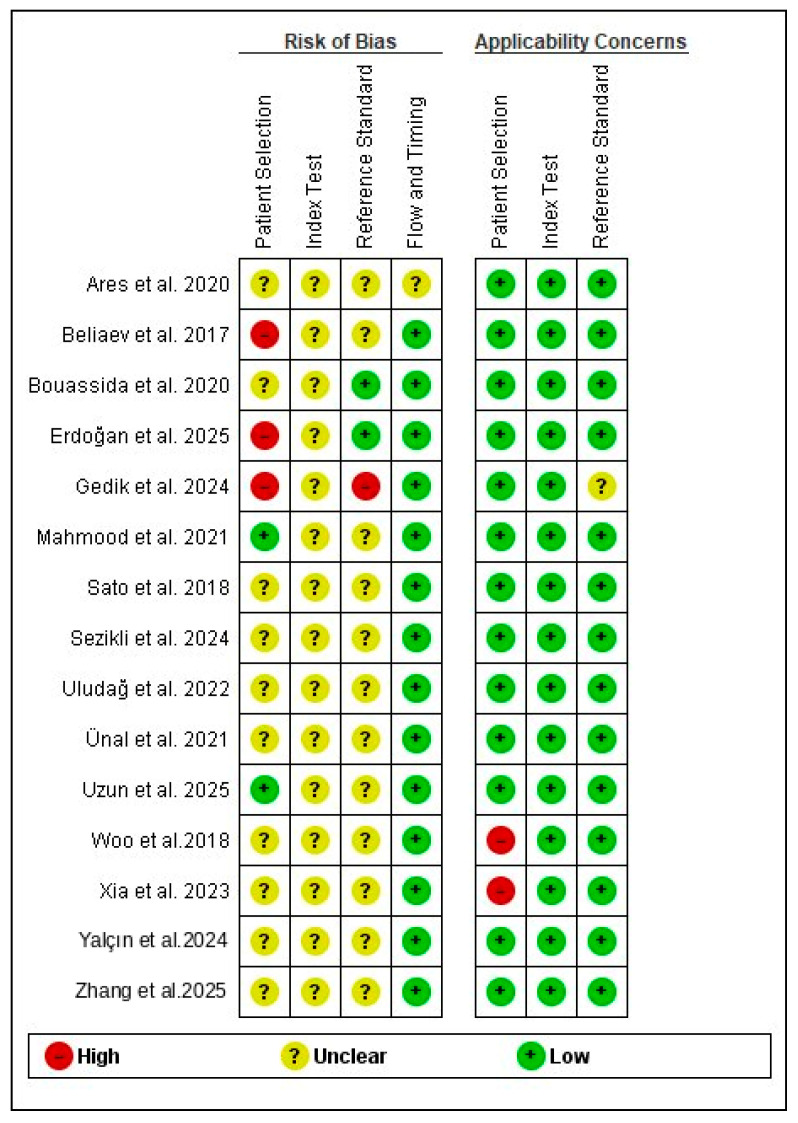
Methodological quality summary [16,17,18,19,20,21,24,25,26,27,28,29,30,31,32].

**Figure 3:**
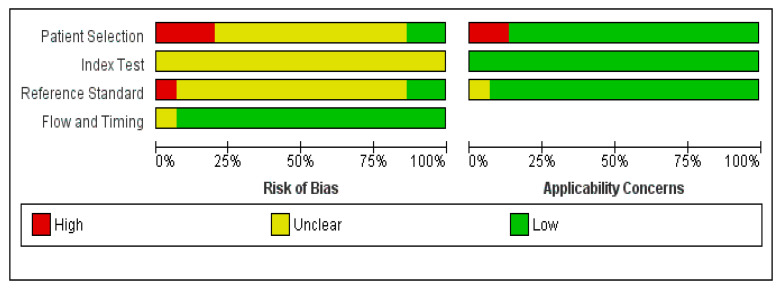
Methodological quality graph.

The quality assessment summary and graph are shown in Figure 2 and Figure 3.

All the included studies had a low risk in the applicability domains except three studies [25,30,31]. Xia et al. and Woo et al. had a high risk due to the inclusion of elderly participants only, while Gedik et al. used clinical diagnosis as a reference standard. Regarding the patient selection domain, only two studies had a low risk in patient selection [19,29]; four studies had a high risk since they included healthy controls [16,18,25,31], while the remaining studies had an unclear risk as they did not provide details on the inclusion of consecutive patients [17,20,21,24,26,27,28,30,32]. All the included studies had an unclear risk in the index domain, since the threshold was not prespecified but was determined according to the ROC curve. Regarding the reference test domain, only Gedik et al. had a high risk since they used clinical diagnosis as a reference standard [25], while Boussida et al. and Erdogan et al. had a low risk [17,18]. The remaining studies had an unclear risk, since there was no clear information on whether the reference tests were interpreted without knowledge of the index test results. Only Ares et al. [24] had an unclear risk in the flow and timing domain, while the remaining studies had low risk of bias.

### 3.4. Outcomes

#### 3.4.1. Diagnosis

The pooled data from three studies [25,29,31] showed that NLR had higher diagnostic accuracy compared to CRP, with a DOR of 2.257 (95% CI 1.1, 4.633). However, there was no significant difference in sensitivity, 0.083 (95% CI −0.018, 0.184), or specificity, −0.010 (95% CI −0.055, 0.034), as shown in Table 2. There was significant heterogeneity for both biomarkers. For CRP, τ^2^ was 0.59 for sensitivity and 3.18 for specificity. For NLR, τ^2^ was 6.46 for sensitivity and 0.51 for specificity. The correlation between sensitivity and specificity was positive for both biomarkers, as shown in Supplementary Table S1.

**Figure 4:**
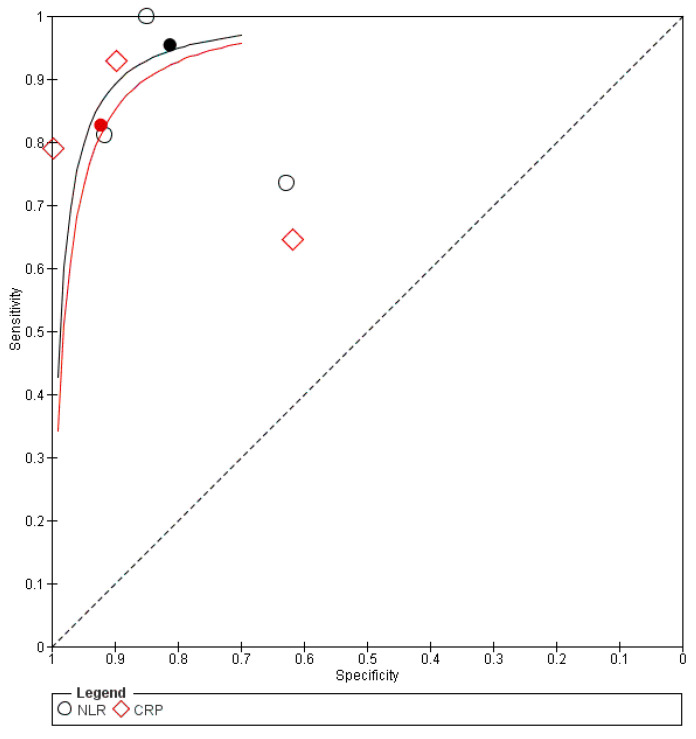
Diagnosis SROC plot. The filled black circle represents the summary operating point. The solid black line represents the SROC curve for NLR; the solid red line represents the SROC curve for CRP; the dashed black line represents the reference line of no discrimination. ○ NLR; ◇ CRP.

The same findings were found after conducting sensitivity analysis, as shown in Supplementary Table S2. The forest plot is shown in Supplementary Figure S1. The SROC curve for NLR was closer to the upper-left corner than that for CRP, thus indicating superior overall diagnostic performance, as shown in Figure 4. A summary of the AUC and corresponding 95% CI is presented in Supplementary Table S3.

#### 3.4.2. Severity According to the Tokyo Guidelines

The pooled data from three studies [18,28,30] showed that NLR had lower overall accuracy compared to CRP in detecting severe cases, DOR 0 0.170 (95% CI 0.081, 0.359). There was no significant difference in sensitivity −0.101 (95% CI −0.214, 0.012), while NLR had higher specificity 0.230 (0.182, 0.279) compared to CRP, as shown in Table 2. For CRP, τ^2^ was 0.51 for sensitivity and 0.17 for specificity. For NLR, τ^2^ was 0 for sensitivity and 0.11 for specificity, as shown in Supplementary Table S1. The correlation between sensitivity and specificity was −1.00 for both biomarkers, likely reflecting instability due to the limited number of included studies.

**Figure 5:**
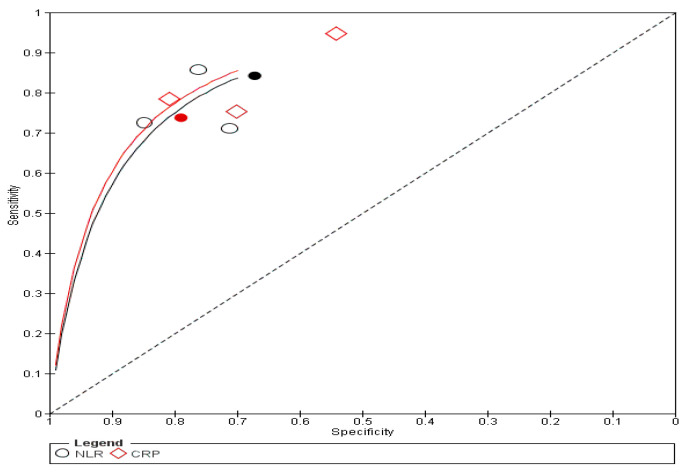
Severity according to TG_SROC plot. The filled black circle represents the summary operating point. The solid black line represents the SROC curve for NLR; the solid red line represents the SROC curve for CRP; the dashed black line represents the reference line of no discrimination. ○ NLR; ◇ CRP.

The sensitivity analysis showed similar results in the overall accuracy and specificity, while NLR had significantly lower sensitivity, as shown in Supplementary Table S2. The forest plot is shown in Supplementary Figure S2. The SROC curve for CRP was closer to the upper-left corner than that for NLR, thus indicating superior overall prognostic performance, as shown in Figure 5. A summary of the AUC and corresponding 95% CI is presented in Supplementary Table S4.

#### 3.4.3. Complications

**Figure 6:**
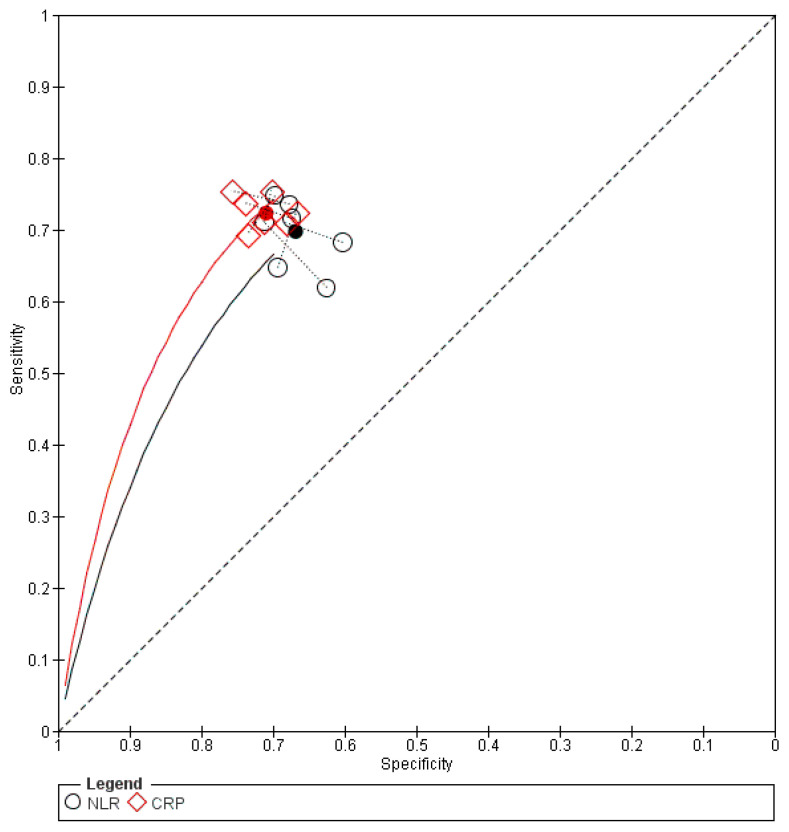
Complications SROC plot.

The pooled data from seven studies [16,19,21,24,27,30,32] showed that there was no significant difference between NLR and CRP in detecting complications, including perforation, gangrene, and suppurative cholecystitis, DOR 1.100 (95% CI 0.817, 1.481). NLR had similar sensitivity −0.029 (95% CI −0.076, 0.019) but lower specificity −0.050 (95% CI −0.090, −0.010) compared to CRP, as shown in Table 2. For CRP, τ^2^ was 0.00 for both sensitivity and specificity, indicating no heterogeneity. For NLR, heterogeneity was low, with τ^2^ values of 0.02 for sensitivity and 0.01 for specificity, as shown in Supplementary Table S1. The correlation between sensitivity and specificity could not be estimated for CRP, while it was 1.00 for NLR, likely due to model instability. The sensitivity analysis showed similar results, as shown in Supplementary Table S2. The forest plot is shown in Supplementary Figure S3. The SROC curve for NLR was characterized by higher summary specificity, while CRP had higher summary sensitivity, as shown in Figure 6. A summary of the AUC and corresponding 95% CI is presented in Supplementary Table S5.

## 4. Discussion

Our systematic review and meta-analysis included 15 studies with 4704 participants. We compared the diagnostic performance of NLR and CRP in the diagnosis and detection of complications and severity grades of patients with acute cholecystitis. We found that NLR had higher accuracy in diagnosing acute cholecystitis compared to CRP. On the other hand, CRP had higher accuracy in differentiating severity grades according to the Tokyo guidelines, while NLR was more specific. Both biomarkers had comparable accuracy in detecting complications such as perforation, gangrene, and suppurative cholecystitis.

Appropriate diagnosis of acute cholecystitis is important since improper diagnosis could have detrimental consequences. For instance, untreated cholecystitis could progress to complications such as gangrene, perforation, and septicemia [33]. We found that NLR had higher diagnostic accuracy compared to CRP. Similar to our study, Kler et al. found that NLR could predict acute cholecystitis [14]. Yet, they investigated the role of NLR only. Nonetheless, among the studies that investigated the NLR and CRP diagnostic roles, four of them compared them to healthy participants [16,18,25,31], while only Uzun et al. [29] included patients who presented with AC and underwent surgery. Similarly, Kler et al. investigated the role of NLR in patients with acute cholecystitis compared to healthy controls [14]. Nonetheless, this does not present real-life scenarios, as patients do not typically present as completely healthy participants. Although we conducted a sensitivity analysis by excluding Uzun et al. [29] and had similar results, this analysis should be interpreted as an exploratory analysis considering the number of included studies.

Appropriate assessment of disease severity is important since it could affect the treatment choice. For instance, patients with mild cholecystitis usually undergo laparoscopic cholecystectomy, while patients with moderate cholecystitis may convert to open surgery. Meanwhile, patients with severe cholecystitis usually undergo conservative treatment and might only undergo surgery under special conditions [34]. Furthermore, Grade III is associated with higher mortality rates compared to Grade I [35]. For instance, Ünal et al. found that mortality rates were 12.5% in grade III, 0.9% in grade II, and 0.2% in grade I [28]. In our study, we found that NLR had lower overall accuracy and sensitivity compared to CRP, while it had higher specificity. This suggests that NLR could be more useful in excluding severe cases. Our results were consistent with the findings of Ünal et al. [28]. In contrast, although Kler et al. found that NLR was significantly higher in patients with severe acute cholecystitis, the NLR cut-off point was not independently associated with severe cholecystitis. Since our analysis included only three studies and the sensitivity analysis showed that NLR had lower sensitivity, these findings should be confirmed through further studies.

The detection of a complicated case of acute cholecystitis is important since it is associated with increased mortality rates [36,37]. However, their detection is challenging using imaging or clinical manifestations since patients could be asymptomatic, while imaging has limited accuracy [7,8,9,10]. Furthermore, although some complications are rare to occur, they could be associated with high mortality rates. For instance, Onder et al. reported a mortality rate of around 18% in patients with gangrenous cholecystitis [38]. Ausania and Date et al. reported mortality rates of 9.5% and 10.8% in patients with gallbladder perforation [37,39]. Meanwhile, mortality could affect up to 70% of patients when diagnosed at late stages [40]. Nonetheless, in our study, we could not assess the impact of each complication separately due to limited data; only one study [21] evaluated the role of NLR and CRP in detecting perforation and found that CRP had a higher AUC of 0.734, while NLR had an AUC of 0.663. However, whether this is statistically or clinically significant is yet to be determined.

We found that both biomarkers had comparable overall accuracy in the prediction of complications such as gangrene, perforation, or suppuration. Yet NLR was significantly less specific. Similarly, Mahmoud et al. found comparable AUC (71.7% for NLR vs. 73.9% for CRP). However, NLR was less specific compared to CRP (66.9% vs. 73.1%) [19]. Arez et al. showed that for the detection of gangrene, CRP had higher accuracy with an AUC of 0.8 compared to 0.75 for NLR. Yet, NLR had a higher sensitivity, 75% compared to 69%, and a lower specificity, 69% compared to 73% in CRP [24]. On the other hand, Uludag et al. found that CRP was more sensitive (72.5% vs. 64.7%) and slightly less specific (66.8% vs. 69.3%) compared to NLR [27]. This could be explained by several factors, including different cut-off points, differences in population characteristics, or differences in detected complications. Among our included studies, two studies exclusively included older-aged patients; although old age is not among the criteria for severity, it indicates a tendency to progress to the severe form [6]. Thus, further studies should explore the role of both biomarkers in elderly patients.

### 4.1. Strengths and Limitations

Our study has several strengths. This is the first systematic review and meta-analysis to compare the diagnostic accuracy of CRP and NLR in patients with acute cholecystitis. We investigated the accuracy of NLR compared to CRP in both the diagnosis and the prognosis of patients with acute cholecystitis. Moreover, we compared the accuracy of both biomarkers in predicting deterioration of the clinical course, as per the Tokyo guidelines. Thus, we addressed several clinical questions. We conducted a primary analysis to investigate which biomarker had superior accuracy using the bivariate model and an additional exploratory sensitivity analysis by excluding studies with outliers such as age, reference tests, or non-healthy controls to confirm the reliability of our findings.

Our study has some limitations. Only five studies evaluated the diagnostic accuracy of NLR and CRP, while the remaining studies predicted the incidence of complications. The included studies involved a variety of controls and reference tests. This heterogeneity might bias the pooled estimates; the inclusion of healthy controls could overestimate specificity, while variation in reference standards might affect sensitivity and the diagnostic odds ratio. Thus, our findings should be interpreted with caution. For instance, Erdogan et al. compared complicated acute cholecystitis to mild cholecystitis or normal participants; thus, we performed a sensitivity analysis and found similar results. There was variability in the age of the included population, and some studies included only older participants, while others included a variable age range. However, given the limited data, we could not conduct a meta-regression to investigate the influence of age. Instead, we performed a sensitivity analysis by excluding Xia et al. and Uzun et al. and found similar results. However, given the limited number of studies, this analysis should be considered as exploratory, and further studies are needed to investigate whether age has an impact on the accuracy of biomarkers. Also, there was heterogeneity in the included studies in the assessment of complications and the reference standard. For instance, some studies used pathological assessment, while others used the clinical course following the Tokyo guidelines or even clinical diagnosis. Thus, we conducted separate analyses for disease severity and another for complications such as gangrene or perforation confirmed through histopathology and we performed a sensitivity analysis by excluding the study that used clinical diagnosis as a reference standard. Nonetheless, we could not assess the accuracy of both biomarkers in detecting each complication separately, such as gangrene, as there was insufficient data. Moreover, several important outcomes, such as perforation and conversion to surgery, were investigated by only one study; thus, they were not meta-analyzed. We included both retrospective and prospective studies; this might introduce bias since retrospective studies are subject to selection bias and might overestimate the diagnostic accuracy. However, given the limited number of studies, we could not perform a subgroup analysis according to the study design. Also, considering the small number of studies included in the diagnosis and severity outcomes, this analysis should be considered as exploratory.

Furthermore, some studies did not mention details on the surgical procedure. Several studies were retrospective and were designed as case–control; nevertheless, in most of them, patients selected as controls were recruited from the same population. Our systematic review included both patients with calculus and acalculus cholecystitis; however, several studies did not report information. Thus, this was not investigated in our meta-analysis. Also, around half of the included studies were conducted in Turkey, which could limit the generalizability of our findings. However, there was insufficient data to perform a subgroup analysis investigating the impact of study settings on the outcomes. None of the included studies pre-specified the threshold for both biomarkers; thus, they had an unclear risk in the index domain. However, this represents the typical approach in diagnostic accuracy studies, where thresholds are frequently determined retrospectively based on the ROC curve. Although CRP and NLR levels may change over time, most studies used a single preoperative measurement obtained upon hospital admission. This reflects real-world clinical decision-making, though biomarker kinetics and symptom duration may influence predictive accuracy.

### 4.2. Clinical Implications

Our systematic review and meta-analysis provided evidence on the diagnostic accuracy of NLR and CRP in the diagnosis and prediction of complications and disease severity according to the Tokyo guidelines. For diagnosis, NLR had higher accuracy compared to CRP, while there was no significant difference in sensitivity or specificity. This suggests that NLR could be used as an adjunctive biomarker in the diagnosis of acute cholecystitis. Nonetheless, the included studies used healthy patients as controls, which does not represent real-world scenarios since patients usually present to the emergency department with acute abdomen rather than being healthy. In clinical practice, the main challenge is differentiating cholecystitis from other causes of acute abdomen. Thus, the diagnostic utility of NLR compared to CRP within a relevant differential diagnosis remains uncertain. Further studies should include patients presenting with acute abdomen to validate these findings in real-world practice. For detecting disease severity, NLR had lower accuracy compared to CRP in detecting disease severity according to the Tokyo guidelines, while it was associated with lower sensitivity and higher specificity. Thus, NLR could be used as a prognostic factor when excluding severe cases, while CRP could be better in screening. Nonetheless, the findings of this study should be interpreted with caution, considering the limited number of included studies. Also, the data showed that there was no significant difference between NLR and CRP in detecting complications, including perforation, gangrene, and suppurative cholecystitis, while NLR had similar sensitivity but lower specificity. Thus, CRP could be used to exclude patients with complications, such as perforation or gangrene. However, the studies investigated various complications. Thus, further studies are needed to investigate each specific complication, such as gangrene or perforation.

## 5. Conclusions

We found that NLR had higher accuracy in the diagnosis of acute cholecystitis compared to CRP, while both biomarkers had comparable sensitivity and specificity. NLR had lower accuracy compared to CRP in detecting disease severity according to the Tokyo guidelines, while it was associated with higher specificity. Meanwhile, NLR and CRP had comparable accuracy and sensitivity in detecting complications such as perforation, gangrene, and suppurative cholecystitis, while NLR had lower specificity compared to CRP. The findings of this study should be interpreted with caution, considering the limited number of included studies and the heterogeneity in the analysis. Further prospective studies are needed to confirm our findings.

## Figures and Tables

**Table 1:** Characteristics of included studies.

Study (Author, Year, Country) Ref.	Study Period	Study Design	*n*	Age, Years Mean (SD)	Male n (%)	Study Population	Calculous AC n (%)	Outcome Assessed	Investigated Condition	Reference Standard
Ares et al., 2020 Spain [24]	January 2016–June 2018	Prospective cohort	180	66.1 (12.33)	90 (50.0)	Patients with AC scheduled for emergency surgery within 24 h of biomarker collection	NR	Complication	Gangrenous cholecystitis	Pathology assessment
Beliaev et al., 2017 New Zealand [16]	May 2004–June 2009	Retrospective cohort	222	60 (12.6)	119 (53.6)	Patients with histologic diagnosis of AC vs. normal gallbladder histology after cholecystectomy	AC: 167 (94.4); Controls: 37 (82.2)	Diagnosis & complication	Acute necrotizing, gangrenous, suppurative cholecystitis; pericholecystic abscess; perforation	Pathology assessment
Bouassida et al., 2020 Tunisia [17]	January 2015–December 2017	Prospective cohort	556	53 (median)	170 (30.6)	Patients with acute calculous cholecystitis undergoing emergency laparoscopic cholecystectomy	556 (100.0)	Complication	Advanced cholecystitis (gangrenous AC, abscess, or peritonitis)	Pathology assessment
Erdoğan et al., 2025 Turkey [18]	September 2019–June 2020	Prospective cohort	56	49.0 (16.3)	35 (62.5)	Adults aged 18–85 with acute gallstone-induced gallbladder inflammation and healthy controls	42 (100.0)	Diagnosis & complication	Severity grades per Tokyo Guidelines (TG18)	Tokyo Guidelines severity grades
Gedik et al., 2024 Turkey [25]	February 2022–May 2022	Prospective cohort	101	NR	NR	Patients aged ≥18 years with no significant comorbidities, diagnosed with acute cholecystitis	NR	Diagnosis	—	Clinical diagnosis
Mahmood et al., 2021 [19] United Kingdom	April 2010–February 2015	Prospective cohort	176	51 (12.7)	58 (33.0)	Patients undergoing emergency laparoscopic cholecystectomy	176 (100.0)	Complication	Gallbladder empyema, necrosis (patchy or complete), gangrene, or perforation	Pathology assessment
Sato et al., 2018 Japan [20]	January 2008–December 2016	Retrospective cohort	262	73 (13.3)	153 (58.4)	Patients with acute cholecystitis	NR	Complication	Severity grades per Tokyo Guidelines (TG18)	Tokyo Guidelines severity grades
Sezikli et al., 2024 Turkey [26]	NR	Retrospective cohort	1352	61.7 (17.2)	560 (41.4)	Patients treated for gallstones with a diagnosis of acute cholecystitis	1352 (100.0)	Complication	Gangrenous cholecystitis	Pathology assessment
Uludağ et al., 2022 Turkey [27]	January 2014–July 2019	Retrospective cohort	250	59.9 (16.6)	121 (48.4)	Patients with AC who underwent cholecystectomy (complicated and uncomplicated)	250 (100.0)	Complication	Gangrenous, perforated, or emphysematous cholecystitis	Pathology assessment
Ünal et al., 2021 Turkey [28]	January 2018–December 2020	Retrospective cohort	528	54.7 (16.8)	205 (38.8)	Patients with acute cholecystitis	NR	Complication	Moderate–severe AC (Grade II or III per Tokyo Guidelines)	Tokyo Guidelines severity grades
Uzun et al., 2025 Turkey [29]	August 2013–August 2023	Retrospective cohort	249	48.9 (14.6)	76 (30.5)	Patients aged ≥17 years with AC in the ED who underwent laparoscopic cholecystectomy within 72 h	249 (100.0)	Diagnosis	—	Pathology assessment
Woo et al., 2018 Korea [30]	January 2012–December 2014	Retrospective cohort	156	76.9 (6.7)	71 (45.5)	Patients aged ≥65 years clinically diagnosed with AC in the emergency department	NR	Complication	Severe AC: gangrenous, necrotizing, suppurative, or perforated cholecystitis (or Grade III per TG)	Pathology assessment or Tokyo Guidelines
Xia et al., 2023 China [31]	December 2018–Nov 2023	Retrospective cohort	220	69.2 (6.2)	99 (31.8)	Patients aged ≥60 years with cholecystolithiasis and cholecystitis who underwent laparoscopic cholecystectomy	160 (100.0)	Diagnosis & complication	Gangrenous and suppurative cholecystitis	Pathology assessment
Yalçin et al., 2024 Turkey [21]	January 2022–August 2023	Case–control	170	58.9 (15.3)	103 (60.6)	Patients presenting to the ED and operated on with a diagnosis of acute cholecystitis	170 (100.0)	Complication	Gallbladder perforation	Pathology assessment
Zhang et al., 2025 China [32]	January 2021–August 2024	Case–control	226	58.8 (14.0)	91 (40.3)	Patients aged ≥18 years with AC who underwent laparoscopic cholecystectomy within 72 h with complete pathology report	0 (0.0)	Complication	Gangrenous cholecystitis	Pathology assessment

**Table 2:** Main analysis.

Outcome	No. of Studies	DOR NLR vs. CRP (95CI)	Difference in Sensitivity NLR—CRP (95CI)	Difference in Specificity NLR—CRP (95CI)
Diagnosis	3	2.257 (1.1, 4.633)	0.083 (−0.018, 0.184)	−0.010 (−0.055, 0.034)
Severity according to TG	3	0.170 (0.081, 0.359)	−0.101 (−0.214, 0.012)	0.230 (0.182, 0.279)
Complications	7	1.100 (0.817, 1.481)	−0.029 (−0.076, 0.019)	−0.050 (−0.090, −0.010)

## Data Availability

The original contributions presented in this study are included in the article/supplementary material. Further inquiries can be directed to the corresponding author.

## Supplementary Materials

The following supporting information can be downloaded at https://www.mdpi.com/article/10.3390/diagnostics16091363/s1, Table S1: Heterogeneity in the analysis. Table S2: Sensitivity analysis. Table S3: Summary of AUC for diagnosis of acute cholecystitis. Table S4: Summary of AUC for detection of acute cholecystitis severity according to Tokyo guidelines. Table S5: Summary of AUC for detection of acute cholecystitis complications. Figure S1: Forest plot showing the sensitivity and specificity of NLR and CRP in diagnosing acute cholecystitis. Figure S2: Forest plot showing the sensitivity and specificity of NLR and CRP in detecting acute cholecystitis severity according to Tokyo guidelines. Figure S3: Forest plot showing the sensitivity and specificity of NLR and CRP in detecting acute cholecystitis complications. File S1: PRISMA 2020 Checklist [41].
