# Supplementary material for: The Diagnostic Accuracy of Neutrophil-to-Lymphocyte Ratio (NLR) Compared to C-Reactive Protein (CRP) in Patients with Acute Cholecystitis: A Systematic Review and Meta-Analysis
Source: Diagnostics (Basel). 2026 Apr 30;16(9):1363. doi: 10.3390/diagnostics16091363 (PMC13163591; doi:10.3390/diagnostics16091363)
Supplement: Supplementary file 1 [file diagnostics-16-01363-s001.zip › diagnostics-4230864-supplementary.docx]

**Search strategy:-**

Search date: 1/2026

**PubMed:**

# (“Acute cholecystitis” OR “acute calculous cholecystitis” OR “acute non-calculous cholecystitis” OR “acute acalculous cholecystitis” OR “Acute Calculus cholecystitis” OR “acute non-Calculus cholecystitis” OR “acute acalculus cholecystitis” OR “Complicated Acute Cholecystitis” OR “Advanced acute cholecystitis” OR “severe acute cholecystitis” OR “suppurative cholecystitis” OR “gallbladder empyema” OR “gangrenous cholecystitis “ OR “gallbladder gangrene” OR ” perforated cholecystitis”) AND (“C-reactive protein” OR CRP OR C-RP) AND (“neutrophil-lymphocyte Ratio” OR NLR OR “neutrophil-to-lymphocyte ratio”)

### Results: 22

### Search fields: All fields

**WOS:**

# (“Acute cholecystitis” OR “acute calculous cholecystitis” OR “acute non-calculous cholecystitis” OR “acute acalculous cholecystitis” OR “Acute Calculus cholecystitis” OR “acute non-Calculus cholecystitis” OR “acute acalculus cholecystitis” OR “Complicated Acute Cholecystitis” OR “Advanced acute cholecystitis” OR “severe acute cholecystitis” OR “suppurative cholecystitis” OR “gallbladder empyema” OR “gangrenous cholecystitis “ OR “gallbladder gangrene” OR ” perforated cholecystitis”) AND (“C-reactive protein” OR CRP OR C-RP) AND (“neutrophil-lymphocyte Ratio” OR NLR OR “neutrophil-to-lymphocyte ratio”)

# Results: 28

Search fields: Topic

**Scopus :**

# (“Acute cholecystitis” OR “acute calculous cholecystitis” OR “acute non-calculous cholecystitis” OR “acute acalculous cholecystitis” OR “Acute Calculus cholecystitis” OR “acute non-Calculus cholecystitis” OR “acute acalculus cholecystitis” OR “Complicated Acute Cholecystitis” OR “Advanced acute cholecystitis” OR “severe acute cholecystitis” OR “suppurative cholecystitis” OR “gallbladder empyema” OR “gangrenous cholecystitis “ OR “gallbladder gangrene” OR ” perforated cholecystitis”) AND (“C-reactive protein” OR CRP OR C-RP) AND (“neutrophil-lymphocyte Ratio” OR NLR OR “neutrophil-to-lymphocyte ratio”)

## Results: 38

Search fields: : TITLE-ABS-KEY

# Table S1. Heterogeneity in the analysis.

| **outcome** | **Biomarker** | **Tau2_Sensitivity** | **Tau2_Specificity** | **Covariance** | **Correlation** |
| --- | --- | --- | --- | --- | --- |
|  |  |  |  |  |  |
| **diagnosis** | **CRP** | 0.59 | 3.18 | 0.41 | 0.30 |
|  | **NLR** | 6.46 | 0.51 | 0.67 | 0.37 |
| **severity according to TG** | **CRP** | 0.51 | 0.17 | -0.29 | -1.00 |
|  | **NLR** | 0.00 | 0.11 | 0.00 | -1.00 |
| **Complications** | **CRP** | 0.00 | 0.00 | 0.00 | Nan |
|  | **NLR** | 0.02 | 0.01 | 0.01 | 1.00 |

# Table S2. Sensitivity analysis.

| **Sensitivity analysis** | | | | | | |
| --- | --- | --- | --- | --- | --- | --- |
| **outcome** | **Exclusion of** | **no. Of studies** | **DOR (95CI)** | **Difference in sensitivity(95CI)** | **Difference in specificity (95% CI)** | **Reason for Exclusion** |
|  |  |  |  |  |  |  |
| **Diagnosis** | Xia et al. 2023 | 2 | 1.341 (0.604, 2.980) | 0.048 (-0.083, 0.178) | -0.005 (-0.052, 0.043) | included older age patients |
|  | Gedik et al. 2024 | 2 | 3.325 (1.307, 8.455) | 0.109 (-0.063, 0.281) | -0.003 (-0.068, 0.062) | compared to a clinical diagnosis as a reference standard |
|  | Uzun et al. | 2 | 2.257 (1.100, 4.633) | 0.083 (-0.018, 0.184) | -0.010 (-0.055, 0.034) | the only study that didn’t include healthy controls |
| **severity according to TG** | Woo etal. 2018 | 2 | 0.078 (0.025, 0.247) | -0.148 (-0.286, -0.01) | 0.277 (0.225, 0.330) | severity according to TG or complications |
|  | Erdogan et al. 2025 | 2 | 0.132 (0.059, 0.294) | -0.129 (-0.252, -0.006) | 0.25 (0.2, 0.3) | compared to normal participants or patients with mild cholecystitis |
| **Complications** | Woo etal. 2018 | 6 | 1.143 (0.838, 1.559) | -0.027 (-0.077, 0.022) | -0.057 (-0.1, -0.015) | severity according to TG or complications |

NLR: Neutrophil-to-lymphocyte ratio. CRP: C-reactive protein. TG: Tokyo guidelines. CI: Confidence interval

# Table S3. Summary of AUC for diagnosis of acute cholecystitis.

| **Study ID** | **index test** | **AUC** | **95CI** |
| --- | --- | --- | --- |
| Erdoğan et al. 2025 | NLR | NR | NR |
|  | CRP | NR | NR |
| Beliaev et al. 2017 | NLR | 0.95 | 0.9-1 |
|  | CRP | 0.97 | 0.93-1 |
| Gedik et al. 2024 | NLR | 0.901 | 0.938-0.964 |
|  | CRP | 0.804 | 0.697-0.911 |
| Uzun et al. 2025 | NLR | 0.716 | 0.628–0.805 |
|  | CRP | 0.655 | 0.553–0.758 |
| Xia et al. 2023 | NLR | 0.9659 | 0.9465–0.9853 |
|  | CRP | 0.9557 | 0.9284–0.9830 |

# Table S4. Summary of AUC for detection of acute cholecystitis severity according to Tokyo guidelines.

| **Study ID** | **index test** | **AUC** | **95CI** |
| --- | --- | --- | --- |
| Erdoğan et al. 2025 | NLR | NR | NR |
|  | CRP | NR | NR |
| Sato et al. 2018 | NLR | 0.729 | 0.657–0.801 |
|  | CRP | 0.744 | 0.675–0.814 |
| Ünal et al. 2021 | NLR | 0.825 | 0.761–0.889 |
|  | CRP | 0.786 | 0.731–0.841 |
| Woo et al. 2018 | NLR | 0.703 | 0.625-0.773 |
|  | CRP | 0.762 | 0.688-0.827 |
| Beliaev et al. 2017 | NLR | 0.99 | 0.97-1 |
|  | CRP | 0.99 | 0.97-1 |

# Table S5. Summary of AUC for detection of acute cholecystitis complications.

| **Study ID** | **index test** | **AUC** | **95CI** | **complication** |
| --- | --- | --- | --- | --- |
| Beliaev et al. 2017 | NLR | 99% | 97%-100% | acute necrotizing, gangrenous, suppurative cholecystitis, and AC associated with pericholecystic abscess and gallbladder perforation |
|  | CRP | 99% | 97%-100% |  |
| Bouassida et al. 2020 | NLR | 0.62 | NR | Advanced cholecystitis (Gangrenous AC, abscess, or peritonitis) |
|  | CRP | 0.75 | NR |  |
| Mahmood et al. 2021 | NLR | 0.746 | 0.655-0.837 | gallbladder empyema, necrosis (patchy or complete), gangrene or perforation |
|  | CRP | 0.773 | 0.698- 0.849 |  |
| Uludağ et al. 2022 | NLR | 0.698 | 0.621–0.775 | Gangrenous, perforated, and emphysema- tous cholecystitis |
|  | CRP | 0.74 | 0.669–0.811 |  |
| Woo et al. 2018 | NLR | 0.703 | 0.625-0.773 | severe AC was defined as the presence of pathologic gangrenous, necrotizing, suppurative, and perforated cholecystitis. If a patient did not have a cholecystectomy, they were classified according to the 2007 Tokyo Guidelines (TG07) for diagnosing severe AC (Grade III). |
|  | CRP | 0.762 | 0.688-0.827 |  |
| Yalcin et al. 2024 | NLR | 0.663 | 0.581 - 0.746 | perforation |
|  | CRP | 0.734 | 0.657 - 0.812 |  |
| Ares et al. 2020 | NLR | 0.75 | NR | gangrene |
|  | CRP | 0.8 | NR |  |
| Sezikli et al. 2024 | NLR | 0.769 | 0.723-0.815 | gangrene |
|  | CRP | 0.616 | 0.561-0.671 |  |
| Zheng et al. 2025 | NLR | 0.748 | 0.686–0.803 | gangrene |
|  | CRP | 0.797 | 0.739–0.847 |  |

---------------------------------------------------------------------------------------------------------------------


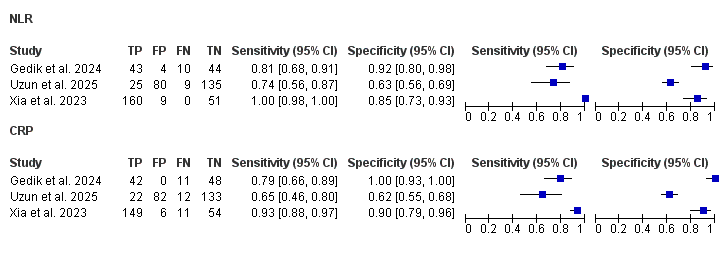


**Figure S1**. Forest plot showing the sensitivity and specificity of NLR and CRP in diagnosing acute cholecystitis.


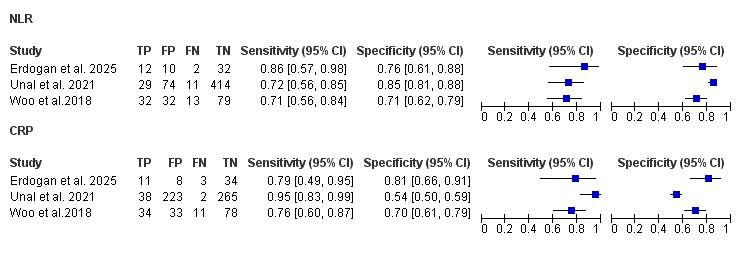


**Figure S2**. Forest plot showing the sensitivity and specificity of NLR and CRP in detecting acute cholecystitis severity according to Tokyo guidelines.


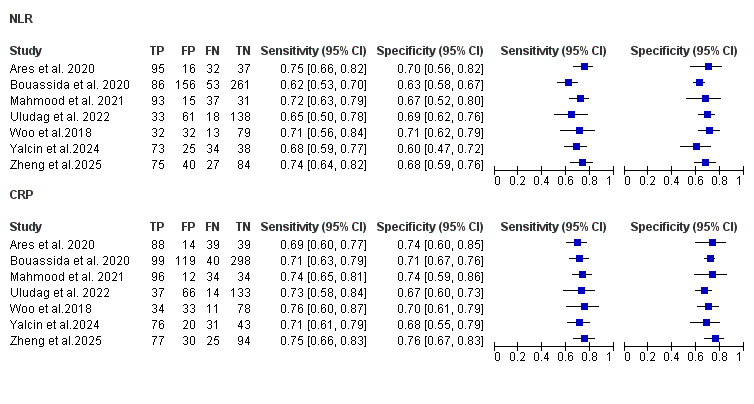


**Figure S3**. Forest plot showing the sensitivity and specificity of NLR and CRP in detecting acute cholecystitis complications.
